# Hand Sanitizer Alert

**DOI:** 10.3201/eid1203.050955

**Published:** 2006-03

**Authors:** Scott A. Reynolds, Foster Levy, Elaine S. Walker

**Affiliations:** * James H. Quillen Veterans Affairs Medical Center, Mountain Home, Tennessee, USA;; † East Tennessee State University, Johnson City, Tennessee, USA

**Keywords:** handwashing, antiseptic, antimicrobial

**To the Editor:** Community-based epidemiologic studies have shown beneficial effects of hand sanitizers. Hand sanitizers were effective in reducing gastrointestinal illnesses in households (*1*), in curbing absentee rates in elementary schools (*2*), and in reducing illnesses in university dormitories (*3*). An Internet search retrieved recommendations for hand hygiene from schools, daycare centers, outdoor guides, and animal shelters.

To reduce infections in healthcare settings, alcohol-based hand sanitizers are recommended as a component of hand hygiene (*4*). For alcohol-based hand sanitizers, the Food and Drug Administration (FDA) (*5*) recommends a concentration of 60% to 95% ethanol or isopropanol, the concentration range of greatest germicidal efficacy. While nonhealthcare groups also recommend alcohol-based hand sanitizers, they usually do not specify an appropriate concentration of alcohol.

Some products marketed to the public as antimicrobial hand sanitizers are not effective in reducing bacterial counts on hands. In the course of a classroom demonstration of the comparative efficacy of hospital-grade antimicrobial soap and alcohol-based sanitizers, a product with 40% ethanol as the active ingredient was purchased at a retail discount store. Despite a label claim of reducing "germs and harmful bacteria" by 99.9%, we observed an apparent increase in the concentration of bacteria in handprints impressed on agar plates after cleansing. None of the other hand cleaners showed such an effect.

Subsequently, we conducted more formal handwashing trials to verify the preliminary finding. Our goal was not to test the products by using the FDA tentative final monograph standard (*5*) but to determine whether a marketed product fails as an antiseptic because of its low alcohol content. To test whether the relatively low concentration of ethanol was the source of treatment failure, we included trials with laboratory-formulated 40% ethanol; we also supplemented the suspect gel with ethanol to a final concentration of 62%. Five hand hygiene treatments were compared: tap water (4 trials), 40% ethanol (5 trials), commercial gels with active ingredients of either 40% or 62% ethanol (9 trials each), and commercial 40% gel supplemented to 62% (5 trials).

At the beginning of each work day, the dominant hand of each volunteer was placed on 150-mm tryptic soy agar plates for 5 s, followed by hand treatment. Each alcohol-based hand treatment involved wetting the hands with 1.5 mL test product followed by vigorously rubbing hands together for 15 s. The tap water treatment differed in that hands were held under running water and vigorously rubbed together for 15 s, followed by air drying. After hands were dry, they were reapplied to a fresh plate for 5 s. Participants were assigned to treatments randomly, but each had to complete each treatment in a week. CFU counts before and after treatment were log transformed to normalize data and compared by using paired *t* tests.

Tap water, 40% ethanol, and 40% ethanol gel yielded no significant reductions in CFU (Table). The 40% gel supplemented with ethanol to a final concentration of 62% reduced the mean CFU by 90%, a level of reduction similar to that of the 62% ethanol gel. Moreover, the 62% gel and the supplemented 40% gel reduced CFU by >50% on all participants. In contrast, only one third of participants showed >50% reductions with 40% gel, one fifth with 40% ethanol, and none with tap water. Differences in pretreatment CFU were not significant (analysis of variance *F* = 1.81, df = 4, 27, p = 0.16). In addition to failing to decrease CFU, colonies were more evenly distributed on postwash plates after use of 40% gel. The even postwash colony distribution may be caused by dispersion of aggregates of microbes without sufficient killing.

**Table:** CFU per plate before and after treatment with various concentrations of ethanol

Treatment	Mean pretreatment CFU (range)	Mean posttreatment CFU (range)	No. trials	|*t*|*	p*	Mean change (%)
Tap water	175 (117–234)	206 (100–321)	4	1.25	0.30	+10
40% ethanol	531 (132–1,413)	621 (75–1,733)	5	0.30	0.39	+3
40% gel	246 (51–602)	232 (56–693)	9	0.61	0.56	+53
62% gel	171 (33–563)	12 (1–24)	9	5.73	<0.001	–82
40%→62% gel	473 (114–1,257)	26 (10–48)	5	6.21	0.003	–90

*|*t*| = result of paired *t* test; p = probability of |*t*|.

Qualitative colony assessment suggested 40% gel and 40% ethanol were as effective as 62% gel against fungi; in contrast, bacterial CFU tended to show little change or increases. The most prevalent bacteria were staphylococci, including those with characteristics of *Staphylococcus aureus*.

After conducting experiments, a survey of 6 local retail chains found no substandard products. In the fall of 2005, a more extensive survey of 18 retail chains (supermarkets, drug stores, general retailers, specialty shops) uncovered a substandard product at all 3 stores of 1 deep-discount chain. The marketing profile of deep-discount chains suggests that poorer segments of the population may be more at risk of purchasing inadequate antiseptic gels. Moreover, 40% ethanol products may be stockpiled in homes and offices. An extensive Internet survey identified no additional substandard commercial products. However, the alcohol content of less-common brands was not always available online, and several Internet sites provide recipes for a bubble gum–scented children's hand sanitizer that contains 33% isopropanol as the sole active ingredient. Educational efforts should emphasize that effective sanitizers must be of a sufficient alcohol concentration.

The efficacy experiments reported here reinforce what has been known for >50 years: 40% ethanol is a less effective bacterial antiseptic than 60% ethanol (*6*). Consumers should be alerted to check the alcohol concentration in hand sanitizers because substandard products may be marketed to the public.
